# Non-invasive cardiac magnetic resonance and electrical myocardial imaging assessment of CRT in patients with heart failure and left bundle branch block

**DOI:** 10.1186/1532-429X-13-S1-O92

**Published:** 2011-02-02

**Authors:** Fady Dawoud, Karl Schuleri, Rozann Hansford, Kristine Evers, B Milan Horacek, Henry Halperin, Albert Lardo

**Affiliations:** 1 Johns Hopkins University, Baltimore, MD, USA; 2 Dalhousie University, Halifax, NS, Canada

## Introduction

CRT is an effective treatment for patients with heart failure and intraventricular conduction delay. However, clinical non-response rates remain unacceptably high; emphasizing the need for improved selection criteria. Central to the underlying mechanism of resynchronization is the relationship between mechanical and electrical activation. The purpose of this study was to demonstrate that mechanical and electrical activation can be quantified in patients with CRT devices combining cardiovascular magnetic resonance (CMR) and electrical myocardial imaging (EMI).

## Purpose

Non-invasively characterize mechanical and electrical activation patterns in patients undergoing cardiac resynchronization therapy (CRT)

## Methods

Patients implanted with biventricular CRT for greater than 9 months (N=10) were mapped with 120 body surface electrodes during both intrinsic activation (device off) and CRT pacing. CMR tagging was also acquired with pacing on/off to perform strain analysis. The circumferential uniformity ratio (CURE) was computed to characterize temporal-spatial mechanical dyssynchrony (value ranging from 0 to 1 where 1 represents perfect synchronous contraction). CMR images of the torso and heart were then used to reconstruct electrical epicardial activation maps using a novel EMI technique. Electrical synchrony was indexed by the mean total activation difference time (ΔMTA, negative values indicate RV activating before LV, on average). Only 2 patients underwent full analysis.

## Results

**Figure 1:**
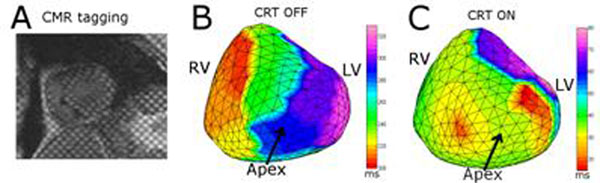
(A)Mid-myocardial, mid-systole short axis MRI tagging image in a patient with CRT device showing sufficient tag quality for strain analysis. (B)Reconstructed electrical activation times during intrinsic activation showing RV preceding LV activation. (C)Synchronized electrical activation of the same patient during biventricular pacing.

CURE decreased from 0.73 to 0.51 when the device was turned off, indicating an increase in mechanical dyssynchrony. The corresponding LVEF decreased as well in on/off modes from 42% to 29%. Figure 1b and 1c show the electrical activation map of the same patient during both modes. Early RV freewall breakthrough consistent with LBBB is seen during pacing off while in the case of biventricular pacing, two areas of early activation (apical RV and basolateral LV) are seen near the tip of the device leads. ΔMTA indicated electrical dyssynchrony without biventricular pacing (-26 ms) as compared to a slight LV earlier activation (9 ms) with pacing.

## Conclusions

CMR and non-invasive EMI are feasible in patients with CRT devices. This ability to quantify mechanical and electrical synchrony before and after CRT may provide insights into mechanisms of resynchronization and lead to robust dyssynchrony metrics that will identify appropriate candidates for CRT.

